# Intensity and Domain-Based 24-Hr Activity Patterns Differ in Their Links to Cognitive and Cardiometabolic Health

**DOI:** 10.1093/geroni/igaf122.1351

**Published:** 2025-12-31

**Authors:** Maddison Mellow, Dorothea Dumuid, Timothy Olds, Ty Stanford, Hannah Keage, Frini Karayanidis, Ashleigh Smith

**Affiliations:** University of South Australia, Adelaide, South Australia, Australia; Alliance for Research in Exercise, Nutrition and Activity (ARENA) Research Centre, University of South Australia, Adelaide, South Australia, Australia; Allied Health and Human Performance, University of South Australia, Adelaide, South Australia, Australia; Alliance for Research in Exercise, Nutrition and Activity (ARENA) Research Centre, University of South Australia, Adelaide, South Australia, Australia; University of South Australia, Adelaide, South Australia, Australia; Functional Neuroimaging Laboratory, University of Newcastle, Newcastle, New South Wales, Australia; Alliance for Research in Exercise, Nutrition and Activity (ARENA) Research Centre, University of South Australia, Adelaide, South Australia, Australia

## Abstract

Each day is made up of a composition of “time-use behaviours.” These can be classified by their intensity (eg, light or moderate–vigorous physical activity [PA]) or domain (eg, chores, socialising). Intensity-based time-use behaviours are linked with cognitive function and cardiometabolic health in older adults, but it is unknown whether these relationships differ depending on the domain (or type/context) of behaviour. This study included 397 older adults (65 ± 3 years, 69% female, 16 ± 3 years education) from Australia. Time-use behaviours were recorded using the Multimedia Activity Recall for Children and Adults (MARCA), cognitive function was measured using the Addenbrooke’s Cognitive Examination III (ACE-III) and Cambridge Neuropsychological Test Automated Battery, and systolic and diastolic blood pressure, total cholesterol, and waist–hip ratio were also recorded. Two 24-hour time-use compositions were derived from each participant’s MARCA, including a 4-part intensity composition (sleep, sedentary behaviour, light, and moderate–vigorous PA) and an 8-part domain composition (Sleep, Self-Care, Chores, Screen Time, Quiet Time, Household Administration, Sport/Exercise, and Social). Linear regressions found significant associations between the domain composition and both ACE-III (p = .010) and waist–hip ratio (p = .009), and between the intensity composition and waist–hip ratio (p = .025). Isotemporal substitution modelling demonstrated that the domains of sedentary behaviours and PA impacted their associations with ACE-III, while any PA appeared beneficial for waist–hip ratio. Findings suggest the domain of behaviour should be considered when aiming to support cognitive function, whereas, for cardiometabolic health, it appears sufficient to promote any type of PA.

